# Derivation and validation phase for the development of clinical prediction rules for rehabilitation in chronic nonspecific low back pain patients: study protocol for a randomized controlled trial

**DOI:** 10.1186/1745-6215-16-4

**Published:** 2015-01-06

**Authors:** Lenie Denteneer, Gaetane Stassijns, Willem De Hertogh, Steven Truijen, Nienke Jansen, Ulrike Van Daele

**Affiliations:** Faculty of Medicine and Health Sciences, University of Antwerp, Universiteitsplein 1, 2610 Wirlijk, Belgium; Department of Physical Medicine and Rehabilitation, Antwerp University Hospital, Universiteitsplein 1, 2610 Wirijk, Belgium; Faculty of Medicine and Pharmacy, Free University of Brussels, Laarbeeklaan 103, 1090 Jette, Belgium

**Keywords:** Clinical prediction rule, Nonspecific, Chronic, Low back pain

## Abstract

**Background:**

There is a consensus that exercise therapy should be used as a therapeutic approach in chronic low back pain (CLBP) but little consensus has been reached about the preferential type of therapy. Due to the heterogeneity of the population no clear effect of specific therapy interventions are found. Probably a specific subgroup of the investigated population will benefit from the intervention and another subgroup will not benefit, looking at the total investigated population no significant effects can be found. Therefore there is a need for the development of clinical prediction rules (CPRs). Objectives for this trial are first, the derivation of CPRs to predict treatment response to three forms of exercise therapy for patients with nonspecific CLBP. Secondly, we aim to validate a CPR for the three forms of exercise therapy for patients with nonspecific CLBP.

**Methods/Design:**

The study design is a randomized controlled trial. Patients with nonspecific CLBP of more than three months duration are recruited at the Antwerp University Hospital (Belgium) and Apra Rehabilitation Hospital. After examination, patients are randomly assigned to one of three intervention groups: motor control therapy, general active exercise therapy and isometric training therapy. All patients will undergo 18 treatment sessions during nine weeks. Measurements will be taken at baseline, nine weeks, six months and at one year. The primary outcome used is the Modified Oswestry Disability Questionnaire score. For each type of exercise therapy a CPR will be derived and validated. For validation, the CPR will be applied to divide each treatment group into two subgroups (matched and unmatched therapy) using the baseline measurements. We predict a better therapeutic effect for matched therapy.

**Discussion:**

A randomized controlled trial has not previously been performed for the development of a CPR for exercise therapy in CLBP patients. Only one CPR was described in a single-arm design for motor control therapy in sub-acute non-radicular LBP patients. In this study, a sufficiently large sample will be included in both the derivation and validation phase.

**Trial registration:**

This trial was registered with Clinicaltrials.gov on 10 February 2014, registration number: NCT02063503.

**Electronic supplementary material:**

The online version of this article (doi:10.1186/1745-6215-16-4) contains supplementary material, which is available to authorized users.

## Background

Low back pain (LBP) can be considered as an epidemic [1]. The European guidelines mention a lifetime prevalence of 84% [2]. About 5 to 10% of LBP patients develop chronic low back pain (CLBP), and 85% of these patients have nonspecific LBP [2]. Based on the European guidelines [2] nonspecific CLBP is defined as pain and discomfort localised below the costal margin and above the inferior gluteal folds, with or without referred leg pain, persisting for at least 12 weeks. Nonspecific LBP is not attributable to a known specific pathology such as infection, tumor, osteoporosis, fracture, structural deformity, inflammatory disorder (for example, ankylosing spondylitis), radicular syndrome or cauda equine syndrome.

The large group of patients meeting these criteria is heterogeneous and therefore these patients represent a treatment challenge for every clinician. There is a consensus that exercise therapy should be used as a therapeutic approach [2] but little consensus has been reached about the preferential type of therapy [3–14]. Due to the heterogeneity of the population no clear effect of specific therapy interventions are found. Probably a specific subgroup of the investigated population will benefit from the intervention and another subgroup will not benefit, looking at the total investigated population no significant effects can be found. In response to this, the European guidelines [2] express the need for the development of tools which improve the classification and identification of specific clinical sub-groups of nonspecific CLBP patients.

Haskins *et al*. [15] described 23 existing studies on clinical prediction rules (CPR) in LBP patients. Only two of those studies describe the development of CPR’s for active rehabilitation in LBP patients. One of those two discusses a McKenzie approach, which is not our point of interest since we are focusing on motor control therapy, general active exercises therapy and isometric training therapy.

The other study, Hicks *et al*. [16], developed two preliminary CPR’s in recurrent LBP patients: one for the success and one for the absence of success of motor control therapy. They performed a single-arm intervention study (no control group only motor control therapy) on 54 recurrent non-radicular LBP patients. After therapy, 39 patients were classified as ‘improved’ and 15 patients were classified as ‘not improved’. Multivariate regression analysis resulted in four positive prediction factors for motor control therapy. Presence of three or more of the predicting factors led to a positive likelihood ratio (LR+) of 4.0 (95% CI: 1.6 to 10.0) and increased the post-test probability of success with motor control therapy from 33 to 67%. Also, four negative predicting factors with motor control therapy were detected. The presence of two or more predicting factors led to a negative likelihood ratio (LR–) of 0.18 (95% CI: 0.08 to 0.38).

Brennan *et al*. [17] investigated the application of CPR’s in 123 acute and sub-acute nonspecific LBP patients based on the CPR by Hicks *et al*. [16] and Fritz *et al.* [18]. The patients that were assigned to an intervention group that, according to the CPR, was not the best suitable therapy (unmatched) were considered as control patients. The patients that were assigned to an intervention group that, according to the CPR, was the best suitable therapy were considered as test patients (matched). They concluded larger improvements on the Modified Oswestry Disability Questionnaire (MODI) by the patients following the matched treatment, in comparison with those following the unmatched therapy, both at short and longer term (four and 52 weeks, respectively).

The implementation and validation of a CPR can lead to a decrease of health complaints and an increased quality of life for numerous patients. A higher number and a faster return to work, or less frequent absence of work over time, are expected. If the patient-customized therapy leads to a decrease of recurrence this can lead to a decrease in direct and indirect health costs.

Our research question is the development (derivation) and validation of a CPR for the choice of an exercise therapy type (motor control therapy, general active exercise therapy and isometric training therapy) in nonspecific CLBP patients. Based on the derived CPR, subgroups will be made, ultimately leading to detection of patients with a high chance for success with a certain type of exercise therapy. The CPR will be developed according to the correct methodological phases [19, 20].

## Methods/Design

Ethical approval (B300201215600) was obtained from the local ethics committees of the University of Antwerp, the Antwerp University Hospital and Apra Rehabilitation Hospital. The trial is registered at Clinicaltrials.gov, with the identification number: NCT02063503.

### Study design

The development of a CPR consists of three consecutive phases. The first two are the derivation and validation phase.

**Figure 1:**
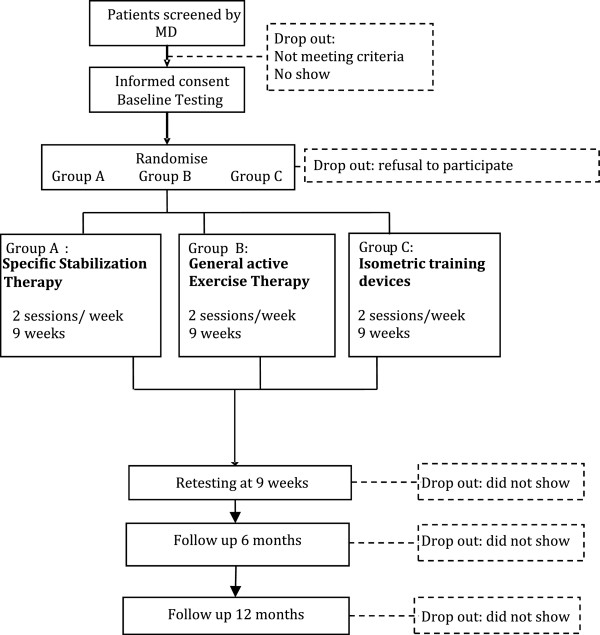
**Flow chart representing the design of the derivation phase.**

In the derivation phase a CPR is derived from a number of variables that have predictive potential for therapy outcome. These variables are obtained from baseline measurements and have a multidimensional character (impairments, activities and/or participation and contextual factors). Data analysis is being used to calculate the most powerful combination of these variables to finally form the new derived CPR (see Figure 1 and ‘Data analysis’).

**Figure 2:**
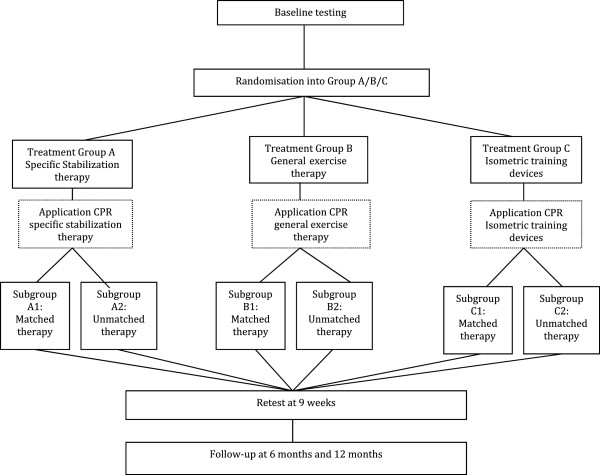
**Flow chart representing the design of the validation phase.**

In the validation phase the derived CPR will be applied in a new study population. This application will result in matched patients (allocated in accordance with the CPR) and in unmatched patients (not in accordance with the CPR). The treatment success of both groups will be compared. The hypothesis is that treatment success will be higher in the matched group (Figure 2).

For both phases, a single blinded randomized controlled trial study design will be used. The project started in August 2014. Analysis of these data is planned for the end 2015.

### Patient recruitment

Patients will be recruited by doctors at the service of two settings located in Antwerp, the Antwerp University Hospital and the Apra Rehabilitation Hospital. Treatment and measurements will be performed at the site where the patient has been recruited.

#### Inclusion criteria

The inclusion criteria for this study are as follows: current nonspecific LBP persisting for at least three months, consulted a medical doctor during the last month because of the persistent LBP, aged between 18 and 65 years, sufficient fluency in Dutch to follow treatment instructions and answer survey questions (Table 1).

**Table 1:** **Inclusion criteria**

Inclusion criteria	Rationale
18 to 65 years old	Chronic low back pain in older adults is more likely to have specific causes (e.g., spinal canal stenosis)
Current nonspecific low back pain persisting ≥ 3 months	Condition studied is specifically chronic
Consulted a doctor during the last month for persistent low back pain	Back pain severe enough and motivation from the patient itself to seek treatment
Dutch fluency sufficient to follow treatment instructions and answer survey questions	Fully informed consent and data collection

#### Exclusion criteria

The exclusion criteria for this study are as follows: spinal canal stenosis, spondylolisthesis and spondylitis, large herniated disc sciatica, radiating pain below the knee, previous back surgery, a history of known spinal fractures, malignancy, known muscle-, nerve-, skin-, or joint diseases, pregnancy and lack of consent (Table 2).

**Table 2:** **Exclusion criteria**

Exclusion criteria	Rationale
Spinal canal stenosis	Back pain possibly due to, specific disease
Spondylolisthesis	
Spondylitis	
Large herniated disc sciatica	
Radiating pain below the knee	
Previous back surgery	
History of vertebral fracture	
Malignancy	
Muscle-, nerve-, skin- or joint diseases	
Known pregnacy	Pregnancy-related low back pain is different in etiology and time course than the target condition for the study (nonspecific chronic low back pain)
Lack of consent	Research policy

### Measurements

#### Baseline testing

The Modified Oswestry Disability Questionnaire (MODI) score will be used as the primary (dependent outcome measure). The MODI is a disease-specific questionnaire to measure disability in LBP patients. The modified version of the Oswestry Disability Questionnaire replaces the sex life item with an employment or homemaking item and has an excellent reliability and good construct validity [21–23]. As secondary (independent) outcome measures the following tests are used:

1. Measurement of impairments: duration of the LBP, pelvis impairments and respiratory impairments (all three items through anamnesis). Prone instability test, straight leg raise, beighton scale, active straight leg raise, sitting knee extension test, waiters bow, pelvic tilt, side support test, extensor endurance test, active sit-up and visual analogue scale for pain (VAS).
2. Measurement of limitations in activities and participation: hours of physical activity per week (trough anamnesis). Short Form 36 Health Survey (SF36), Roland Morris Disability Questionnaire (RMDQ).
3. Measurement of contextual factors: gender, age, height, weight, body mass index, smoking, profession, underwent previous therapy and comorbidity (trough anamnesis), Tampa scale for kinesiophobia and Fear Avoidance Belief Questionnaire (FABQ),

We performed a literature search to select all used clinical tests based on their reliability and validity [24]. Further, clinical tests used by Hicks *et al*. [16] were also included in our study design. The baseline testing takes about 30 minutes.

#### Follow-up

Short-term follow-up will take place after completion of the treatment program at nine weeks. All primary and secondary outcome measures from the baseline testing will be reevaluated at this time point.

Long-term follow-up will take place at six months and one year after entering the study. The following outcome measurements will be inventoried: age, weight, hours of physical activity per week, profession, duration of LBP, MODI, Tampa scale for kinesiophobia and VAS. The patients will initially be contacted by mail, and by phone if they do not respond to the mail within two weeks to make an appointment (Table 3).

**Table 3:** **primary (dependent) and secondary (independent) outcome**

Measures	0 weeks	9 weeks	6 months	1 year
***Primary outcome:***				
MODI	X	X	X	X
***Secondary outcome***				
***Impairments:***				
Duration low back pain*	X	X	X	X
Pelvis impairments*	X	X		
Respiratory impairments*	X	X		
PIT	X	X		
SLR	X	X		
Beighton scale	X	X		
ASLR	X	X		
SKET	X	X		
Waiters bow	X	X		
Pelvic tilt	X	X		
Side support test	X	X		
Extensor endurance test	X	X		
Active sit up	X	X		
VAS*	X	X	X	X
***Secondary outcome***				
***Activities and participation:***				
RMDQ	X	X		
SF-36	X	X		
Hours physical activity/week*	X	X	X	X
***Secondary outcome***				
***contextual factors:***				
Tampa scale	X	X	X	X
FABQ	X	X		
Gender*	X			
Age*	X	X	X	X
Height*	X			
Weight*	X	X	X	X
BMI*	X	X		
Smoking*	X	X		
Profession*	X	X	X	X
Previous therapy*	X			
Comorbidity*	X	X		

*Information obtained through anamnesis.

*MODI: modified oswestry disability questionnaire, PIT: prone instability test, SLR: straight leg raise, ASLR: active straight leg raise, SKET: sitting knee extension test, VAS: visual analogue scale, RMDQ: roland morris disability questionnaire, FABQ: fear avoidance belief questionnaire, BMI: body mass index.*

### Randomization and blinding

In both phases patients will be randomly assigned to one of the three treatment groups after baseline testing. The responsible researcher will use a randomization list generated with Microsoft Excel® software (version 14.3.9, Microsoft Corporation, Zaventem, Belgium). Physiotherapists responsible for the treatment sessions are blinded for the results of baseline and follow-up measurements, which are being performed by the responsible researcher.In the validation phase a pre stratification will be used to ensure a 50:50 ratio between matched and unmatched therapy within one intervention group. Patients who have a strong preference for one of our intervention groups and therefore cannot be randomized will be asked to participate in a parallel cohort study. In this study similar baseline and follow-up measurements will be used.

### Intervention

During the nine-week intervention, patients will be treated two times a week. Patients will be assigned randomly into one of three intervention groups (motor control therapy, general active exercise therapy and isometric training therapy). Each intervention will take about 70 minutes. A 10 minutes warm up and cool down period will be the same in each treatment regime. Each group receives 50 minutes of therapy-specific intervention. Previously trained physiotherapists will give the treatment. To ensure that all therapists provide the same exercises, a treatment protocol for each treatment group was developed and a treatment diary will be filled out after each session. Interventions in the derivation and validation phase are similar.

#### Motor control therapy

The local stabilizing muscles (such as the multifidus, transversus abdominis, pelvic floor muscles and diaphragm) together with the global muscles (such as the erector spinae and rectus abdominis) are important in creating spinal stability [25–27]. An impaired spinal stability is considered an important factor in developing LBP [28–30]. This leads to the conceptualization of motor control therapy for the lumbar spine. Such therapy involves very specific low-load exercises of deeper trunk muscles that are dysfunctional in nonspecific CLBP [31, 32] and has proven to be effective in nonspecific LBP [4–7, 10].

Patients in this intervention group follow motor control training based on the work by Richardson and Hides [33]. There are three levels to be completed by the patients. To evolve to the next level the patient must be able to correctly execute each exercise described in the former level. However, if the patient is not able to do so in the first level within three weeks of treatment or after six sessions, we will automatically proceed to the second level with the intention to minimalize the drop out and maximize the patient’s compliance. The first level is based on the isolated contraction of the local motor control muscles. Contraction of the transversus abdominis is performed with low intensity controlled by palpation. The second level is based on the contraction of local and global motor control and global mobilizing muscles. In the third level patients will practice the contraction of local and global motor control and global mobilizing muscles in functional patient specific conditions (for example a patient who has to perform a lot of loaded trunk rotations during work will get specific exercises in a rotation movement direction, similar to the work condition) (Tables 4 and 5).

**Table 4:** **exercise summary**

Treatment regimens:	Motor control therapy	General active exercise therapy	Isometric training therapy
***First phase MCT:***			
ADI in supine position	X		
ADI in sitting position	X		
ADI in sitting position 1DTM	X		
ADI in high squat position	X		
ADI in medium squat position	X		
ADI in supine position with heel lifts			
ADI in supine position with heelslide	X		
ADI with superman exercise	X		
ADI with top leg turn out	X		
***Second phase MCT:***			
ADI with side bridging	X		
ADI with back bridging	X		
ADI in sitting position 3DTM	X		
Hip extension	X		
Side-squat	X		
Reverse lunge	X		
Star excursion exercise	X		
***Third phase MCT:***			
ADI in functional movements	X		
Leg-press		X	
Leg-extension		X	
Calf raise		X	
Standing abduction		X	
Standing adduction		X	
Lat pull down		X	
Low row		X	
Triceps		X	
Biceps		X	
Chest press		X	
Shoulder press		X	
Fly		X	
Front raise		X	
Side raise		X	
Active sit up		X	
Hamstring stretch		X	
Quadriceps stretch		X	
Adductor stretch		X	
Latissimus dorsi stretch		X	
Trapezius stretch		X	
TD for lumbar flexion			X
TD for lumbar extension			X
TD for lumbar rotation			X
TD for lumbar lateroflexion			X
Stationary bike	X	X	X

*MCT: motor control therapy, ADI: abdominal drawing in, DTM: dimensional trunk movements, TD: Tergumed device.*

**Table 5:** **exercise modalities**

Treatment regimens:	Motor control therapy	General active exercise therapy	Isometric training therapy
Total intervention time	70 minutes	70 minutes	70 minutes
Warming up	10 minutes	10 minutes	10 minutes
Cooling down	10 minutes	10 minutes	10 minutes
Regime specific intervention time	50 minutes	50 minutes	50 minutes
Intensity	Low load	60% 1RM	30-40% MIS
Number of sets	2	3	2
Number of repetitions	20	20	20
Duration of one repetition	6 seconds	-	5 seconds
Rest between sets	30 seconds	30 seconds	30 seconds

*1RM: one repetition maximum, MIS: maximal isometric strength.*

#### General active exercise therapy

General active therapy has proven to be an effective therapeutic approach in nonspecific LBP [5, 6, 14]. These patients receive a global form of exercise therapy existing of all kinds of active exercises. These exercises do not have the direct purpose of contracting the multifidus or transversus abdominis. Aerobic conditioning exercises will be performed for lower and upper limbs, strength training of the back and abdominal muscles and stretching (Tables 4 and 5).

#### Isometric training therapy

Effectiveness of isometric training therapy has been proven in nonspecific LBP [11–13]. The Tergumed™ (version 1.0, Enraf-Nonius NV, Boom, Belgium) training protocol is followed. It contains progressive resistance isometric training of lumbar trunk muscles on devices in the directions flexion, extension, rotation and lateral flexion. In the first session, the maximal isometric strength is determined in all movement directions to set the level of resistance for training. During the first to fourth week, patients train at 30%, and during the fifth till ninth week, at 40% of their maximal isometric strength. When the difference in maximal isometric power for rotation or lateral flexion direction between abdominal and back muscles exceeds 20%, the weaker side has to perform five sets and the stronger side has to perform four sets (Tables 4 and 5). Exercises mentioned in Table 4 are further explained in Additional file 1, which explains the performance of each exercise.

### Power analysis

#### Derivation phase

As we cannot predict the therapeutic effect in the derivation phase, we use a two-tailed hypothesis to calculate the power. A pilot study showed that the standard deviation of our primary outcome measure, the MODI, is comparable to the results of Brennan *et al*. [17]. Given these estimates, 120 patients are needed to detect a minimum clinically important difference (effect size 0.43) with 80% power using a two-tailed hypothesis. This means that three groups of 40 patients are needed to complete the whole trial. Since Van der Wouden *et al*. [34] mentioned a dropout rate of 54%, we aim to include 260 patients.

#### Validation phase

In the validation phase the intervention therapy stays the same, but new patients will be included. In this study design, the interest is that treatment success will be higher in the matched group than the unmatched group, which means a one-tailed hypothesis is used to calculate the power. In the validation phase we need 105 patients to detect a minimum clinically important difference (effect size 0.43) with 80% power using a one-tailed hypothesis. This means that three groups of 35 patients are needed to complete the whole trial. Again we assume a dropout rate of 54% into account and therefore aim to include 229 patients.

### Data analysis

#### Derivation phase

This phase aims to detect potential predictive variables for treatment success (dichotomous, yes or no) in a set of baseline measurements. A minimal decrease in the MODI score of six points is considered as treatment success [35].

Potential predictive variables are selected as follows: first, individual variables from the self-reports, history and physical examination are tested for their bivariate association with the reference standard using independent sample t tests, Mann-Whitney U tests or chi-square tests based on the nature of the data. Variables with a significance level of *P* <0.10 are retained as potential prediction variables. We chose a more liberal significance level at this stage to avoid excluding potential predictive variables.

Next, selected potential prediction variables and known predictors as determined in the study of Hicks *et al*. [16] are entered into a multiple linear regression equation to determine the optimal set of predictors. A significance of *P <*0.05 is used in this stage. Variables retained in the regression model are used to develop a multiple CPR for classifying patients as likely responders to treatment. This statistical analysis is performed for each treatment group (motor control therapy, general active exercise therapy and isometric training therapy).

#### Validation phase

In the validation phase new patients will be again assigned randomly into one of the three treatment groups. Based on the derived CPR determined in phase one, each intervention group will be divided into either ‘matched therapy’ or ‘unmatched therapy’. The recruitment, information form, informed consent, measurements, blinding and intervention procedure will be the same for both derivation and validation phase.

To verify intergroup differences at baseline, variables will be compared between groups using independent t tests, Mann-Whitney U tests, or chi-square tests of independence based on the nature of the data.

To examine the principle hypothesis (difference in treatment effect between matched and unmatched groups), a three-way repeated-measures analysis of variance will be performed with treatment group (motor control versus general active exercise therapy vs. isometric therapy) and classification subgroup (motor control versus general active exercise therapy versus isometric therapy) as between-subject variables and time (baseline, nine weeks, six months and one year) as the within-subject variable. The dependent variable is disability (MODI score). The hypothesis of interest is the three-way interaction, and the two-way interactions between time and treatment group and between time and classification subgroup. We hypothesize that outcome over time will not differ based on the randomized treatment group, or the classification subgroup, but will depend on the interaction between the treatment group and classification subgroup, such that patients randomized to matched treatment will have better outcomes than patients randomized to unmatched treatment. This hypothesis will be supported if the three-way interaction is significant, but the two-way interactions are not. Pairwise *post-hoc* comparisons will be performed at each follow-up period to further explore any significant interaction terms. In both the derivation and validation phase we will use an intention-to-treat analysis.

### Information form and informed consent

If patients meet the inclusion and exclusion criteria they are scheduled for an interview with one of our researchers to be informed about the trial. If patients decide to participate they will sign an informed consent. Information form and informed consent are made and have been approved by the ethics committee of the University of Antwerp, the Antwerp University Hospital and Apra Rehabilitation Hospital.

## Discussion

The goal of this trial is the derivation and validation of a CPR for the choice of an exercise therapy type in nonspecific CLBP patients. Our study protocol differs from previous studies on several points.

First, a randomized clinical trial with several treatment options is used. So far, only one CPR for motor control therapy is derived in patients with non-radicular LBP [16]. A single-arm intervention study design was used. The derivation of a CPR for exercise therapy should be repeated in a randomized controlled trial design to confirm and reinforce findings. If a randomized controlled trial design is used, differences between different intervention groups can be made, and patients are randomly allocated into these groups to ensure there is no difference between the groups for success outcome.

Second, we target CLBP patients with a sufficiently long history of LBP. Hicks *et al*.’s [16] study sample had a mean duration of current symptoms for 40.6 days. Patients with symptoms for 40.6 days are classified as having sub-acute LBP [2]. Brennan *et al*.’s [17] study sample had a mean duration of current symptoms for 16 days, which are acute and sub-acute LBP problems [2]. In contrast, we aim to include patients with persisting LBP for at least three months to ensure we only target genuine CLBP patients [2]. We target CLBP patients because these patients are a big challenge for adequate treatment and they bring high health-related costs to society. Since both studies describe an acute and sub-acute study sample, our study results might differ from theirs [16, 17]. In conclusion we state that derivation, validation and impact phase of the development of a CPR for exercise therapy in a chronic LBP study sample has not yet been described in the literature.

Third, we aim to include a sufficiently large sample, based on a power analysis. Hicks *et al*. [16] included 54 patients in their study but no power analysis is mentioned in their paper. If there are not enough patients included, the CPR is unlikely to be valid. We compared our calculated sample size with the sample size calculated in Brennan *et al*.’s [17] study design and found comparable results. In addition, we use an extra 54% inclusion target to compensate for the possible dropout rate [34]. We aim to recruit 489 patients in total (combined total for both phases of the study). We calculated that each year around 200 LBP patients start the back revalidation program in the Antwerp University Hospital. This is the same for the Apra Rehabilitation Hospital. Out of these 400 LBP patients around 50% have nonspecific complaints. This means that we need around 18 months to finish each phase. We will closely follow the inclusion procedure to make sure we meet our goals. The current study protocol describes only the derivation and validation phase, and not the impact phase. Future research should target this impact phase. The impact phase needs a completely different approach since it investigates the economic consequences of the implication of the CPR. It also depends on the outcome of the derivation and validation phase. Consequently, it is beyond the scope of the present paper.

## Trial status

This study is currently recruiting patients. The project started in August 2014. As we need around 18 months to finish each phase, we expect the study to finish end 2017.

### Consent

Written informed consent was obtained from the patient(s) for publication of this manuscript and accompanying images. A copy of the written consent is available for review by the Editor-in-Chief of this journal.

## Electronic supplementary material

Additional file 1: **Performance exercises Table 4.** (PDF 574 KB)

## Abbreviations

CI: Confidence interval
CLBP: Chronic low back pain
CPR: Clinical prediction rule
FABQ: Fear Avoidance Believe Questionnaire
LBP: Low back pain
LR: Likelihood ratio
MODI: Modified Oswestry Disability Questionnaire
RMDQ: Roland Morris Disability Questionnaire
SF36: Short Form 36 Health Survey
VAS: Visual Analogue Scale.
