# Supplementary material for: Derivation and validation phase for the development of clinical prediction rules for rehabilitation in chronic nonspecific low back pain patients: study protocol for a randomized controlled trial
Source: Trials. 2015 Jan 6;16:4. doi: 10.1186/1745-6215-16-4 (PMC4326449; doi:10.1186/1745-6215-16-4)
Supplement: Supplementary file 1 — Additional file 1: Performance exercises Table 4. (PDF 574 KB) [file 13063_2014_2391_MOESM1_ESM.pdf]

Additional file 1: Performance exercises table 4

Exercise	Performance	Visualization
Abdominal drawing in (ADI) in supine position	Patient lies supine. Knees are flexed with feet supported at hip width.<br>Patient and/or therapist palpate contraction of the m. transversus abdominis. Patient keeps breathing during contraction.	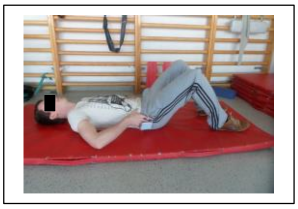
ADI in sitting position	Patient is seated on a bench.<br>Patient and/or therapist palpate contraction of the m. transversus abdominis. Patient keeps breathing during contraction.	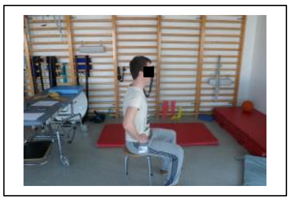
ADI in sitting position 1 dimensional trunk movements	Patient is seated on a bench.<br>Patient contracts m. transversus abdominis. During contraction patient executes hip flexion followed by hip extension. The lumbar spine stays in a neutral position.	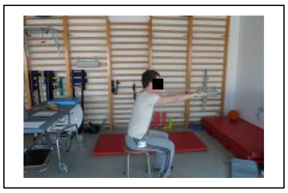
ADI in high squat position	Patient is standing. Feet are positioned at hip width. Patient contracts m. transversus abdominis. During contraction patient executes a high squat. Knees and hip joints flex 30°.	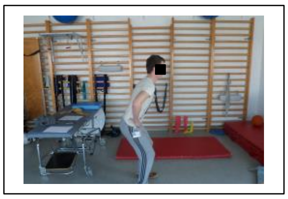
ADI in medium squat position	Patient is standing. Feet are positioned at hip width. Patient contracts m. transversus abdominis. During contraction patient executes a medium squat. Knees and hip joints flex 60°.	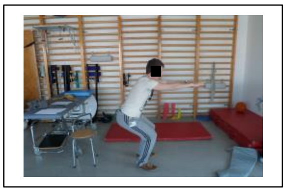
ADI in supine position with heel lifts	Patent lies supine. Knees are flexed with feet supported at hip width.<br>Patient contracts m. transversus abdominis. During contraction patient lifts one foot and extends the leg. The lifted foot does not touch the ground during the exercise. Exercise is executed left and right.	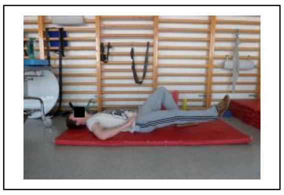
ADI in supine position with heelslide	Patient lies supine. Knees are flexed with feet supported at hip width.<br>Patient contracts m. transversus abdominis. During contraction patient extends one leg. The heel of the extended leg keeps in contact with the ground.<br>Exercise is executed left and right.	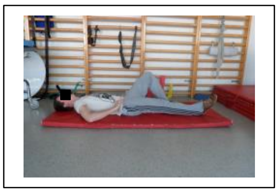

ADI with superman exercise	Patient is positioned on knees and hands.<br>Patient contracts m. transversus abdominis. During contraction patient extends one leg and the opposite arm. The lumbar spine stays in a neutral position.<br>Exercise is executed left and right.	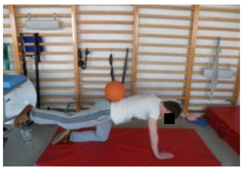
ADI with top leg turn out	Patient lies in side position. Elbow is positioned under the head. Both legs are flexed at 90°. Patient should maintain the body position in one line.<br>Patient contracts m. transversus abdominis. During contraction patient executes a 45° abduction and exorotation of the upper leg. Both feet are kept together. Exercise is executed left and right.	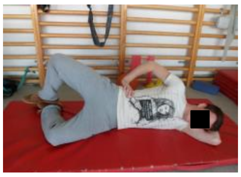
ADI with side bridging	Patient lies in side position**.** Elbow is flexed 90° and supports the body weight. Both legs are flexed 90°.<br>Patient should maintain the body position in one line.<br>Patient contracts m. transversus abdominis. During contraction patient lifts pelvis from the ground. The body should be positioned in one line during execution. Exercise is executed left and right.	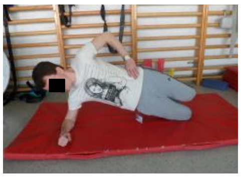
ADI with back bridging	Patient lies supine. Knees are flexed with feet supported at hip width.<br>Patient contracts m. transversus abdominis. During contraction patient lifts pelvis from the ground. The body should be positioned in one line during execution.	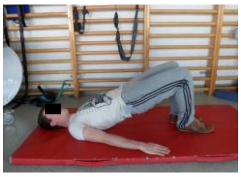
ADI in sitting position 3 dimension trunk movements	Patient is seated on a bench.<br>Patient contracts m. transversus abdominis. During contraction patient executes 3D flexion and extension movements of the lumbar spine.	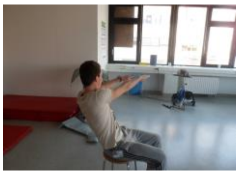
Hip extension	Patient is standing and supports with hands against the wall.<br>Patient contracts m. transversus abdominis. During contraction patient executes a hip extension of one leg. The body position maintains an upright position. The lumbar spine stays in a neutral position. A Thera-band can be used for extra resistance.	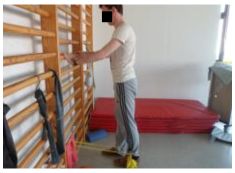
Side-squat	Patient is standing. Feet are positioned at hip width.<br>Patient contracts m. transversus abdominis. During contraction patient moves one leg laterally and executes a side squat movement. Exercise is executed left and right.	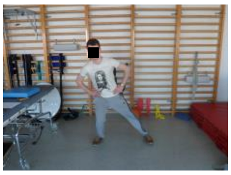

Reverse lunge	Patient is standing. Feet are positioned at hip width. Patient contracts m. transversus abdominis. During contraction patient moves one leg backwards. Hips are lowered to the floor until front knee forms a 90° angle. Exercise is executed left and right.	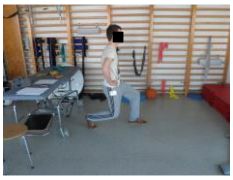
Star excursion exercise	Patient is standing on one leg.<br>Patient contracts m. transversus abdominis. During contraction patient moves the contralateral leg as far as possible in 8 different directions (star form).<br>Exercise is executed left and right.	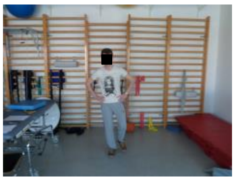
Leg-press	Patient is seated. Knees are flexed and at hip width. Patient extends knees and uses both feet to push weight away.	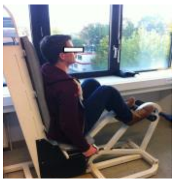
Leg-extension	Patient is seated.<br>Patient executes a knee extension movement against the resistance. Exercise is executed left and right.	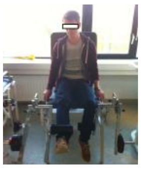
Calf raise	Patient is standing and supports with hands against the wall bars.<br>Patient raises heels and extends ankles as high as possible.	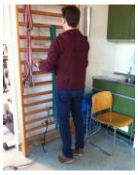
Standing abduction	Patient is standing and supports with one hand against the wall bars.<br>Patient executes an abduction movement of one leg.<br>A thera-band is used to adjust the resistance.<br>Exercise is executed left and right.	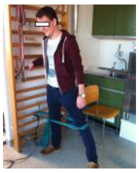
Standing adduction	Patient is standing and supports with one hand against the wall bars.<br>Patient executes an adduction movement of one leg.<br>A thera-band is used to adjust the resistance.<br>Exercise is executed left and right.	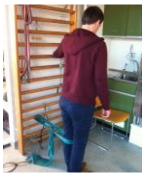
Lateral pull down	Patient is seated. Arms are held overhead at full extension, grasping a bar connected to the weight stack.<br>Patient pulls the bar down towards the chest.	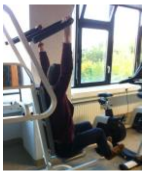

Low row	Patient is seated.<br>A Thera-band is attached to the wall bars. Patient grasps the Thera-band and initiates the exercise with arms extended. Patient executes shoulder retroflexion and elbow flexion	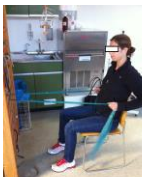
Triceps	Patient is seated.<br>A Thera-band is attached to the wall bars. Patient grasps the Thera-band and initiates the exercise with elbows flexed. Patient executes elbow extension.	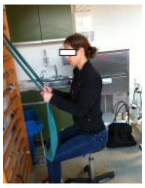
Biceps	Patient is seated.<br>A Thera-band is attached to the wall bars. Patient grasps the Thera-band and initiates the exercise with elbows extended. Patient executes elbow flexion.	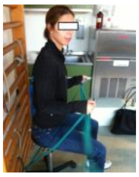
Chest press	Patient is seated.<br>A Thera-band is attached to the wall bars. Patient grasps the Thera-band and initiates the exercise with 90° shoulder abduction and 90° elbow flexion. Patient maintains shoulder position and executes an elbow extension.	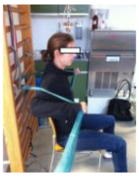
Shoulder press	Patient is seated.<br>A Thera-band is attached to the wall bars. Patient grasps the Thera-band and initiates the exercise with 90° shoulder abduction and exorotation. Elbows are 90° flexed and palms of hands are facing forward. Patient extends elbows and hands move upward.	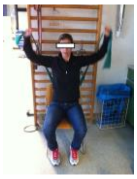
Fly	Patient is seated.<br>A Thera-band is attached to the wall bars. Patient grasps the Thera-band and initiates the exercise with 90° shoulder abduction and full elbow extension. Patient maintains elbow extension and moves hands towards the middle line.	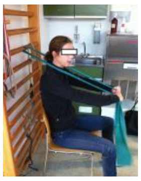
Front raise	Patient is seated.<br>A Thera-band is attached to the wall bars. Patient grasps the Thera-band and initiates the exercise with a neutral shoulder position and full elbow extension. Patient maintains elbow extension and performs a 90° shoulder elevation.	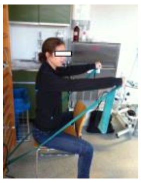
Side raise	Patient is seated.<br>A Thera-band is attached to the wall bars. Patient grasps the Thera-band and initiates the exercise with a neutral shoulder position and full elbow extension. Patient maintains elbow extension and performs a 90° shoulder abduction.	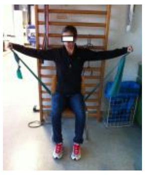

Active sit up	Patient lies supine. Knees are flexed with feet supported at hip width. Arms are extended. Patient contracts abdominal muscles and moves upward until both scapulae are lifted from the ground.	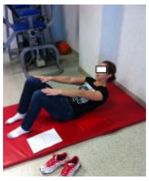
Hamstring stretch	Patient stands with one leg just in front of the other. The back knee is flexed. Patient should bent forward from the hips and sit back into the stretch until stretch is felt.	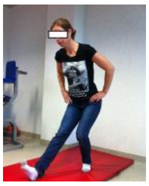
Quadriceps stretch	Patient lies in side position. The lower leg is flexed to keep the lumbar spine in a flexed position. Patient grasps ankle of upper leg and executes a knee flexion and hip extension until stretch is felt.	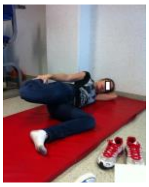
Adductor stretch	Patient is standing with feet wide apart and extended knees. Patient bends knee to one side and leans towards the same side until stretch is felt. Exercise is executed left and right.	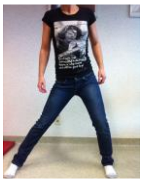
Latissimus dorsi stretch	Patient is standing with feet wide apart and extended knees. Patient moves one arm above the head and executes a lateroflexion movement away from the elevated arm until stretch is felt. Exercise is executed left and right.	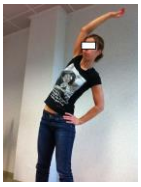
Trapezius stretch	Patient is seated with both legs extended. Patient sits on one hand to maintain shoulder depression. Patient uses opposite hand to pull the head into a lateroflexion position until stretch is felt. Exercise is executed left and right.	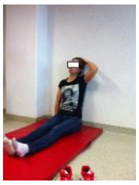
Tergumed device for lumbar flexion	Patient is seated. The Tergumed device is set to the body proportions of the individual patient. Patient executes a lumbar flexion movement.	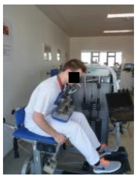
Tergumed device for lumbar extension	Patient is seated. The Tergumed device is set to the body proportions of the individual patient. Patient executes a lumbar extension movement.	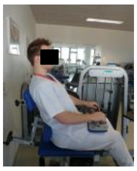

Tergumed device for lumbar rotation	Patient is seated.<br>The Tergumed device is set to the body proportions of the individual patient. Patient executes a lumbar rotation movement. Exercise is executed left and right.	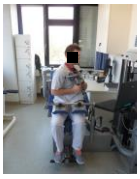
Tergumed device for lumbar lateroflexion	Patient is seated.<br>The Tergumed device is set to the body proportions of the individual patient. Patient executes a lumbar lateroflexion movement. Exercise is executed left and right.	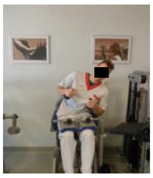
